# Brazilian study of adaptation and psychometric properties of the Coping Health Inventory for Parents

**DOI:** 10.1186/s41155-017-0065-9

**Published:** 2017-05-11

**Authors:** Regina Basso Zanon, Mônia Aparecida Da Silva, Euclides José De Mendonça Filho, Denise Ruschel Bandeira, Manoel Antônio Dos Santos, Ricardo Halpern, Cleonice Alves Bosa

**Affiliations:** 1 Postgraduate Program in Psychology of the Federal University of Rio Grande do Sul, Rua Ramiro Barcelos, 2600 - Térreo, Porto Alegre, 90035-003 Brazil; 2 Department of Philosophy, Sciences and Letters of the Ribeirão Preto College of the University of São Paulo, Av. Bandeirantes, 3900 - Monte Alegre, Ribeirão Preto, SP 14049-900 Brazil; 3 Federal University for Medical Sciences of Porto Alegre, Rua Sarmento Leite, 245, Porto Alegre, RS 90050-170 Brazil

**Keywords:** Coping, Parents, Measurement, Test validity, Rasch analysis

## Abstract

The Coping Health Inventory for Parents (CHIP) evaluates coping patterns of parents of chronically ill children and assesses different coping strategies using three subscales. This study aimed to translate and transculturally adapt the CHIP for a Brazilian sample and investigate the preliminary psychometrics of the scale. Rating scale Rasch analysis was performed on CHIP responses, and the psychometric performance of each of the three subscales was tested. Two hundred twenty parents of individuals with health problems participated in the study, answering a sociodemographic questionnaire—the Brazilian version of the CHIP—and Folkman and Lazarus’s coping questionnaire. All items exhibited good fit to the measurement model, although response categories were not used as intended and little variability on person parameter estimates was obtained. These preliminary results suggested that each construct being measured by the three subscales should be treated separately, corroborating the theoretical model of the original instrument. Suggestions to address the psychometric limitations of the instrument were made in order to improve measurement precision.

**Electronic supplementary material:**

The online version of this article (doi:10.1186/s41155-017-0065-9) contains supplementary material, which is available to authorized users.

## Background

The diagnosis of a health condition in a child imposes new responsibilities and challenges on the parents. There is evidence that the parents feel they are not prepared to deal with the new situation in the family and, as a result, they seem to become more vulnerable to stress factors (Cantekin et al. 2015; Favero-Nunes and Santos 2010). In fact, studies show that changes in the family routine, frequent in this context, can wear on the emotional, physical, and social stability of the caregivers, which can trigger psychopathologies such as stress, depression, and anxiety disorders (Chamak and Bonniau 2013; Favero-Nunes and Santos 2010; Zablotsky et al. 2013). In turn, the impact of these symptoms on the parents can, among other complications, influence the quality of care provided to the children and the manner in which they connect, if at all, to the available health services (e.g., Zand et al. 2013).

Meanwhile, when attempting to cope with their child’s health problems, the parents frequently develop strategies for reducing tension and anxiety, which can (or not) contribute to the process of adaptation to the demands of a chronic illness. In this regard, the literature identifies as protective factors the quality of coping strategies employed and familial relationships and the social support received (Hebert and Koulouglioti 2010; Lai et al. 2015; Lai and Oei 2014; Zablotsky et al. 2013).

Traditionally, coping strategies have come to be defined as behavioral or cognitive efforts used by people to deal with stressful situations (Lazarus and Folkman 1984). Operationally, a coping strategy is treated as a cognitive or behavioral response to the stress, undertaken with the goal of reducing the adverse effect of perceived stress. In fact, research has shown that the use of adaptive coping strategies correlates positively with well-being and negatively with stress (e.g., Benson 2014). Beyond this, studies reveal that in the context of families of children with chronic illnesses or with health problems requiring periodic interventions, those strategies can vary according to parental personality traits, education, and gender as well as to the child’s diagnosis and age (Lai and Oei 2014).

Various instruments are used throughout the world for evaluating specific coping strategies, for example, *The Mainz Coping Inventory* (Krohne et al. 2000), the *Coping Strategy Indicator*, and *The Measure of Daily Coping* (Stone and Neale 1984), among others. However, currently, there is only one scale adapted for a Brazilian sample: Folkman and Lazarus’s Ways of Coping checklist (Folkman and Lazarus 1985; Savóia et al. 1996), which consists of a questionnaire of 66 items that encompass the thoughts and actions people use to deal with the internal or external demands of a specific stressful event. This questionnaire has been much used in the Brazilian literature (e.g., Araujo et al. 2010); however, it is not designed for use with families of people with health problems, in particular those requiring continuous and specialized care. With this in mind, the *Coping Health Inventory for Parents* (CHIP) (McCubbin, 1987) was developed to evaluate coping patterns specifically in the health care context. It is a 45-item checklist providing self-reported information about how parents perceived their overall response to the management of family life with a chronically ill child. It has three subscales: (a) family (family integration, cooperation, and optimistic definition of the situation); (b) support (maintaining social support, self-esteem, and psychological stability); and (c) medical (understanding the medical situation through communication with other parents and consultation with the medical professionals).

The CHIP has been administered in various international studies in order to measure coping patterns of parents of individuals with different diagnoses and health conditions, such as cancer, Prader-Willi syndrome, autism spectrum disorders, and asthma (Garro 2011; Lakkis et al. 2015; Tvrdik et al. 2015). Considering the paucity of instruments for investigating parental coping specifically in the area of health care in Brazil and the importance of understanding the means by which such strategies aid the process of the parents’ adaptation to the child’s chronic or acute illness, the present study sought to translate and culturally adapt the CHIP for a Brazilian sample, as well as to investigate the preliminary psychometric properties of the scale. More specifically, an investigation of the rating scale via Rasch modeling and internal consistency of the scales as well as evidence of convergent validity was undertaken.

## Methods

### Participants

Two hundred twenty fathers and mothers participated in the study. All of them were parents of children (0 to 27 years old) with a health problem for whom some sort of specialized care was needed in private and/or public health care services. Children’s health problems included some kind of deficiency (e.g., autism spectrum disorder (ASD), intellectual disability), chronic illness (e.g., asthma, meningitis), or acute illness (e.g., intestinal infection).

The sample was selected by convenience and by snowballing (Silverman et al. 1990). It was treated as a mixed collection, involving both online and in-person interaction. Contact with the participants took place in hospitals, special schools, and associations for families of children with deficiencies. In the face-to-face data collection, the parents received physical copies of the instruments, while for the online collection, the questionnaires were made available via the SurveyMonkey® platform. The order in which the instruments were presented was the same for both collection types. The in-person parents represented 77.7% (*n* = 171) of the sample, while the online group was 22.3% of it (*n* = 49).

### Instruments

The sociodemographic questionnaire, designed specifically for the present study, consisted of 25 objective questions that covered information such as education, occupational and marital status, and religion.

The final version of the CHIP in Portuguese that went through the process of translation and transcultural adaptation for a Brazilian test sample is available in Additional file 1. The items which constitute the instrument are scored against a Likert scale, which ranges from 0 to 3, with 0 being “not helpful” and 3 being “extremely helpful.” Additionally, there was an option for the respondents to indicate “chose not to use it” or “not possible.” It should be emphasized that these response options were excluded from the analysis and considered as missing data. The original version of the scale has three dimensions (i.e., maintaining family integration, cooperation, and an optimistic definition of the situation; maintaining social support, self-esteem, and psychological stability; and understanding the medical situation through communication with other parents and consultation with medical staff). In this study, these dimensions will be called family, support, and medical, respectively. The internal consistency of these three subscales in the original version of the instrument was satisfactory, with Cronbach’s alphas of .79, .79, and .71, respectively (McCubbin 1987). With regard to the translation and transcultural adaptation process, it is emphasized that the content of all the items was preserved in the simple translation from English to Portuguese. In the next step, a back-translation from a bilingual translator, blind to the original version, was examined by two independent referees regarding the semantic equivalence and cultural suitability. Differences between the two versions were reconciled by consensus, resulting in the final version, which was approved by the author of the original version. The Ways of Coping checklist of Folkman and Lazarus (Folkman and Lazarus 1985) was used in the analysis of the external validity of the CHIP. In that instrument, the questions are scored on a Likert scale as follows: 0 (I did not use this strategy); 1 (I used it occasionally); 2 (I used it often); and 3 (I used it a lot). The scale has eight factors proposed by the authors which were preserved in the factorial analysis in the Brazilian sample. They are the following: confrontation (factor 1); distancing (factor 2); self-control (factor 3); social support (factor 4); accepting responsibility (factor 5); escape-avoidance (factor 6); problem-solving (factor 7); and positive reappraisal (factor 8). The instrument was translated and adapted to Portuguese by Savóia et al. (1996). The authors analyzed the concurrent, factorial, and internal validity of the instrument for a Brazilian sample. To evaluate the internal validity, the authors utilized the method of halves, which obtained a correlation between the total scores of the test and retest of *r* = .704. Through factorial analysis, using principal factor analysis with oblique rotation, the researchers found eight factors that corresponded to the coping pattern scales from the original study (and explained 70.8% of the total variance).

### Procedures

Initially, the process of translating and transculturally adapting the CHIP for a Brazilian sample was developed according to the methodological recommendations proposed by Borsa et al. (2012) and involved the following steps: (1) translation of the instrument to the new language, done by two independent bilingual translators; (2) synthesis of the two translated versions, done by the team of researchers after comparing the different translations and evaluating their semantic, idiomatic, conceptual, linguistic, and contextual discrepancies; (3) evaluation of the synthesis by experts, in this case by a specialist in the area of psychological evaluation; (4) evaluation by the target audience, composed of five parents of children with autism spectrum disorders, with the goal of verifying that the items, the instructions, and the answer scale were understandable by the population for which the instrument was intended; (5) back-translation, done by two independent bilingual translators not involved in the forward-translation and presentation of the synthesis of the two versions to the original author of the study; and (6) a pilot study done with a small but representative sample of the target population.

### Data analysis procedures

To evaluate the psychometric properties of CHIP, the rating scale model of Andrich (1978), an extension of the dichotomous Rasch Model (1960; Golino et al. 2015), for polytomous items was used in each of the subscales. This procedure estimates the location of people in a latent continuum as result of participants’ responses following the probability of a specified response (e.g., I did not use this strategy/I used it occasionally/I used it often and I used it a lot) modeled as a function of person and item parameters (Bond and Fox 2015). This model can independently assess the item difficulty parameters (*δ*) and latent trait level of a sample of individuals in the same linear continuum. Results are expressed as log-odds units (logits) in which both parameters are in a same metric.

The fit of items to the measurement method was assessed with infit mean-square and outfit mean-square residual indexes. The infit mean-square is an information-weighted measure of item fit, in which is more sensitive to the pattern of responses that are close to the item difficulty discrepancies. The outfit is not an information-weighted measure of item fit, so it is more sensitive to responses of people that are far from the difficulty of the item. According to, outfit mean-square problems are less of a threat to measurement than the infit mean-square. As much in the case of infit as outfit, items with indexes near 1.0 display good fit (Linacre 2002). Thus, the infit mean-square and outfit mean-square values considered a recommended interval of 0.50 to 1.50 (Linacre 2002), with values closer to 1.0 indicating a better explanation by the model and values above 1.50 as degrading the measurement system.

The model assumptions of unidimensionality and local independence were assessed through principal component analysis of residuals and residual correlations of items. For unidimensionality, items with factor loadings bigger than |0.4| on the residual correlation where considered meaningful for another factor. For local dependence, values above |.30| were considered evidence of local dependence (Bond and Fox 2015).

Person and item reliability coefficients were used as an indicator of true variance and error variance. Its interpretation is similar to the popular estimator of raw-score reliability, the Cronbach’s Alpha, with the advance that for inference beyond the test, Rasch reliability is more conservative and less misleading (Linacre 1997). Person and item separation indexes were also considered; these indexes are estimates of the sample’s spread relative to the precision (SE) of those measurements. Low person separation, smaller than 2, implies that the instrument may not be sensitive enough to distinguish between high and low performers, suggesting that more items may be needed, whereas low item separation (smaller than 3) implies that the person sample is not large enough to confirm the item difficulty hierarchy (Linacre 2010a, 2010b).

To investigate the external validity, the Spearman correlations were calculated between the CHIP person parameters with Folkman and Lazarus’s Ways of Coping checklist scores. The Spearman correlation was used in recognition of the asymmetric distribution of the data.

The analyses were performed using the Statistical Package for the Social Sciences and Winsteps® (Linacre 2010a, 2010b).

## Results

Table 1 presents the main sociodemographic characteristics of the participants. The majority of the respondents were female (82.3%, *n* = 181) and lived in the South and Southeast Brazil (74.1%, *n* = 108). The average age of the participants was 36.24 years, (*SD* = 7.78). The monthly income of parents (in BRL, Brazilian Reais) was R$ 3635.01 (*SD* = 2622.70), the equivalent of US$ 1112 (*SD* = 802.69), using exchange rate at the time of the study (August 2016). Regarding the education level of the parents, the majority had completed secondary school or higher (74.1%, *n* = 163).

**Table 1:** Sociodemographic characteristics of the participants (*n* = 220)

Characteristic	Percent	Number
Gender		
Female	82.3	181
Male	17.3	38
No answer	0.4	1
Education		
1st to 3rd grade of primary school	1.8	4
4th to 7th grade of primary school	9.5	21
Completed primary school	5.5	11
Some secondary school	8.6	19
Completed secondary school	27.3	60
Some college	15.0	33
Completed college	16.8	37
Graduate degree (MS, PhD, etc.)	15.0	33
No answer	0.5	1
Marital status		
Married or with a partner	75.0	165
Single	14.5	32
Widowed	1.4	3
Divorced	9.1	20
Marital satisfaction		
Extremely unsatisfied	4.1	9
Unsatisfied	3.6	8
Indifferent	5.5	12
Satisfied	41.4	91
Extremely satisfied	28.6	63
No answer	16.8	37
Has a job		
Yes	69.0	152
No	21.9	28
No answer	9.1	20
Has religious/spiritual belief		
Yes	91.4	201
No	8.1	18
No answer	0.5	1
Actively practices spiritual/religious beliefs		
Yes, always	27.3	60
Yes, sometimes	33.2	73
Yes, rarely	10.5	23
No	16.4	36
No answer	12.6	28
Caring for more than one ill family member		
Yes	10.0	22
No	89.1	196
No answer	0.9	2

The majority of participating parents were married or lived with a partner (75.0%, *n* = 165) and most (*n* = 183) were satisfied or higher with the marriage (*n* = 154, 84.2%). Sixty-nine percent of the participants (*n* = 152) had a job, and most (89.1%, *n* = 196) cared for their own child only. A great many of the parents declared themselves to be religious or spiritual (91.4%, *n* = 201), and of these, 27.3% (*n* = 60) were actively engaged in their religions.

Table 2 presents the sociodemographic characteristics and development of the children of the sample participants. The average age of the children was 7.55 years (*SD* = 4.74), ranging from 0 to 27, and majority of them were female and lived with their mother and father. The majority had a chronic illness of organic origin or some type of deficiency (i.e., physical disability, sensory impairment, intellectual disability, or ASD). Regarding education, the largest group was in pre-school. The majority had required some kind of intensive medical care in the previous year.

**Table 2:** Sociodemographic characteristics and development of the children with health problems (*n* = 220)

Characteristic	Percent	Number
Gender		
Female	60.0	132
Male	38.6	85
No answer	1.4	3
Medical condition		
Deficiency (physical, sensory, intellectual, or ASD)	40.8	90
Chronic organic illness	42.8	94
Acute organic illness	16.4	36
Lives with		
Mother and father	53.2	117
Mother, father, and other family	21.8	48
Only with mother	21.8	48
Other	3.2	7
Education		
Never went to school	15.5	34
Pre-school	37.3	82
1st to 3rd grade	19.5	43
4th to 7th grade	17.6	39
Completed primary school	2.3	5
Some secondary school	2.7	6
Completed secondary school	1.4	3
Some college	0.5	1
Completed college	0.5	1
No answer	2.7	6
Attending school		
Yes	79.1	174
No	20.4	45
No answer	0.5	1
Receiving specialized care		
Yes	66.8	147
No	33.2	73
Required intensive medical care in the last year		
Yes	40.4	89
No	59.1	130
No answer	0.5	1
Health care status		
Public	49.1	108
Private	17.7	39
Public and private	28.2	62
Not applicable	2.7	6
No answer	2.3	5

### Psychometric properties

From the preliminary analysis performed by Rasch using the rating scale model, there was evidence that the subjects did not make use of all the response categories as intended. The rating scale model presupposes that, along the latent characteristic, each response category has a distinct probability of being chosen more than any other category for a specific item. For example, for item 3, not one person chose 0 (“not helpful”), which demonstrates that the probability of this response category being chosen across the latent characteristic will always be less than the alternatives. Therefore, the four alternative responses of the original instrument were recategorized into three categories. The alternatives 0 (“not helpful”) and 1 (“minimally helpful”) were combined into one response category while the other two original alternatives, “moderately helpful” and “extremely helpful,” remained in their original categories. This procedure was also performed in a previous study that validated the CHIP (Gothwal et al. 2015).

After recoding, all scales exhibited good item reliability indexes indicating that each item’s variance was modeled by the latent trait and not by measurement error. Item separation indexes were also satisfactory, confirming that the obtained item hierarchy was robustly estimated. All of the items were fit to a measurement model. For the family scale, the model was able to explain 30% of the response variance. Two items (Chip4 and Chip5) violated the local dependence assumption and had factor loadings above .5 and residual correlation of .72. The support scale had 40% of its variance explained by the model and two items (Chip23 and Chip24) had significant factor loadings at the residual principal component analysis and a high residual correlation of .71. Finally, the medical scale had 50% of its variance explained by the rating scale model, items Chip42 and Chip44 factor loadings of .46 and .60, respectively, but weak local dependence with a correlation of .28.

**Fig. 1:**
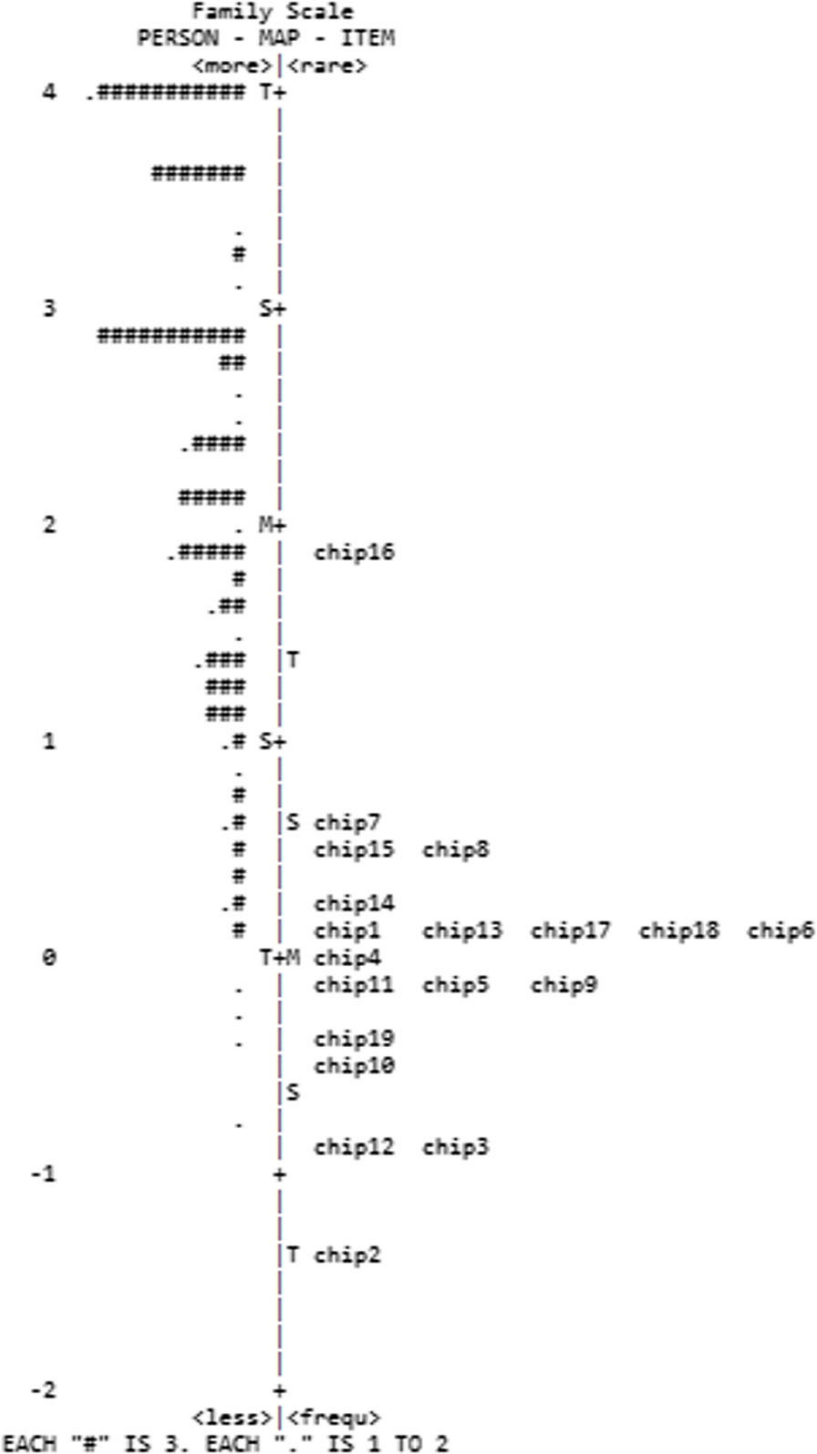
Item-person map for the family dimension of the CHIP

**Fig. 2:**
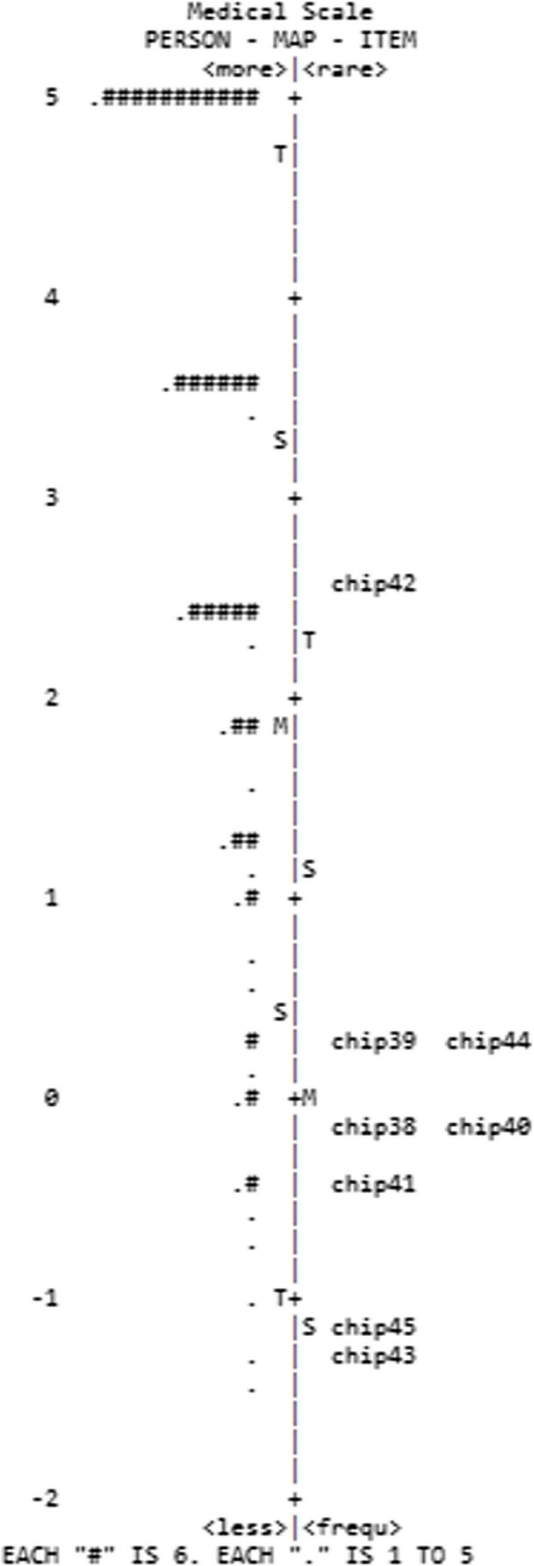
Item-person map for the support dimension of the CHIP

**Fig. 3:**
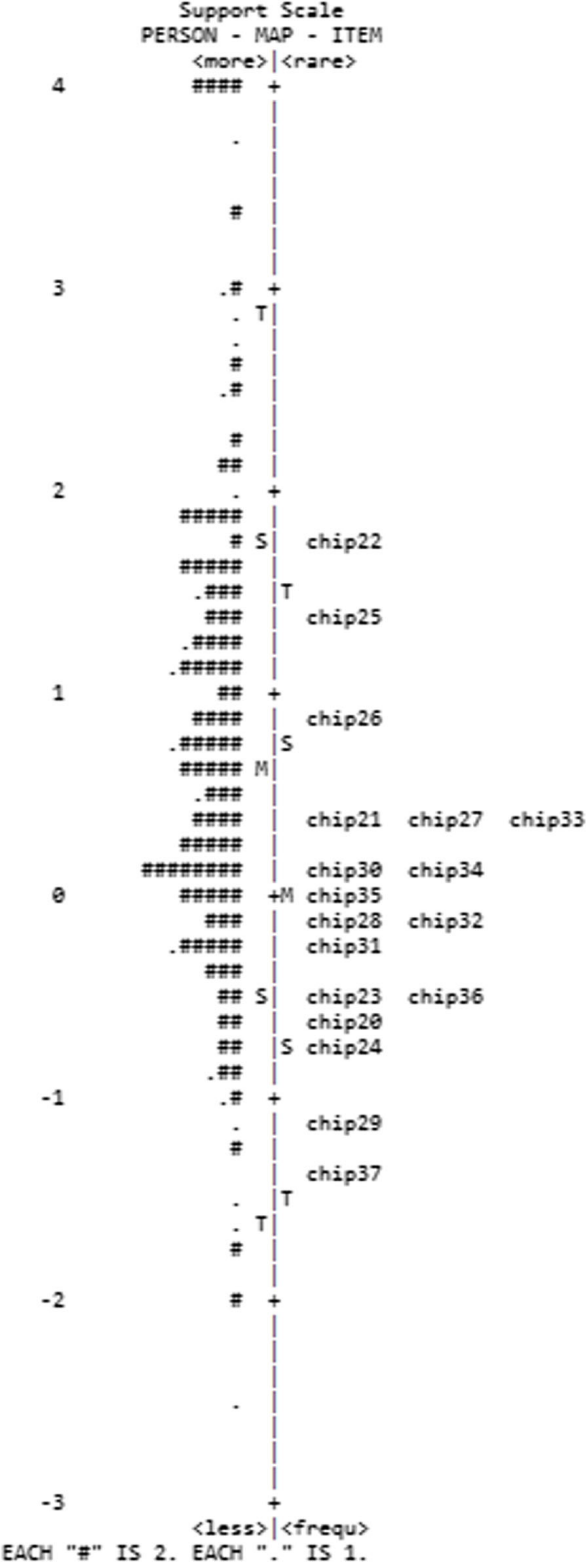
Item-person map for the medical dimension of the CHIP

According to Table 3, the family and medical scales had very low person reliability and separation indexes. The item-person map displays the localization of the item parameters, as well as the distribution of the person parameters, along the latent trait. These figures are useful for comparing the range and position of the distribution of the items (right panel) with the position of the latent trait of the people (left panel). Ideally, the items should be located along the whole scale to precisely measure the intended construct (Mair et al. 2015). In accordance with the item-person map of the family and medical dimensions (Figs. 1 and 2), it is worth noting that the distribution of the participants’ scores was high and asymmetric, with very easy items not ranging over the total extent of the scale. The items and person parameters of the support scale were better distributed along the latent scale, and thus, a greater capacity for discriminating the participants throughout the latent continuum is observed in Fig. 3.

**Table 3:** Parameters and fit of items to the measurement models for each CHIP subscale (*n* = 220)

	Item	Δ	Infit	Outfit	Error	Polarity
Family	Chip1	0.14	1.13	1.16	0.15	0.42
	Chip2	−1.38	0.93	1.13	0.25	0.3
	Chip3	−0.82	0.99	1.11	0.18	0.39
	Chip4	−0.04	1.06	1.46	0.16	0.4
	Chip5	−0.18	0.97	0.55	0.17	0.42
	Chip6	0.14	1.1	0.93	0.16	0.46
	Chip7	0.66	1.01	0.95	0.14	0.56
	Chip8	0.52	0.95	0.9	0.14	0.54
	Chip9	−0.18	1.03	0.84	0.18	0.35
	Chip10	−0.5	0.91	0.78	0.18	0.43
	Chip11	−0.08	0.68	0.57	0.16	0.55
	Chip12	−0.88	0.76	0.49	0.19	0.52
	Chip13	0.09	0.9	0.84	0.15	0.53
	Chip14	0.26	1.18	0.96	0.15	0.45
	Chip15	0.44	1.22	1.51	0.14	0.39
	Chip16	1.85	1.31	1.26	0.12	0.53
	Chip17	0.13	0.97	1.15	0.15	0.46
	Chip18	0.14	0.98	0.89	0.15	0.47
	Chip19	−0.32	1.15	1.17	0.18	0.33
Item reliability			0.94			
Person reliability			0.60			
Item separation			3.84			
Person separation			1.22			
Support	Chip20	−0.59	1.04	0.95	0.12	0.5
	Chip21	0.32	1.14	1.13	0.11	0.5
	Chip22	1.74	1.18	1.2	0.13	0.54
	Chip23	−0.53	1.01	1.05	0.12	0.49
	Chip24	−0.7	1	1.25	0.12	0.44
	Chip25	1.32	1.26	1.26	0.12	0.51
	Chip26	0.93	0.82	0.82	0.11	0.68
	Chip27	0.37	0.89	0.9	0.11	0.61
	Chip28	−0.15	1.2	1.38	0.11	0.42
	Chip29	−1.1	0.89	0.97	0.13	0.48
	Chip30	0.16	0.98	0.97	0.11	0.57
	Chip31	−0.23	1.13	1.04	0.12	0.49
	Chip32	−0.1	0.94	0.89	0.11	0.58
	Chip33	0.35	0.87	0.83	0.11	0.63
	Chip34	0.12	1.07	1.11	0.12	0.51
	Chip35	−0.03	0.91	0.89	0.12	0.59
	Chip36	−0.46	0.87	0.9	0.12	0.57
	Chip37	−1.41	0.96	0.85	0.14	0.47
Item reliability			0.98			
Person reliability			0.83			
Item Separation			6.37			
Person separation			2.22			
Medical	Chip38	−0.12	0.76	0.7	0.17	0.66
	Chip39	0.22	1.14	1.1	0.17	0.62
	Chip40	−0.09	0.74	0.62	0.17	0.68
	Chip41	−0.42	0.93	1.05	0.18	0.58
	Chip42	2.62	1.33	1.39	0.15	0.75
	Chip43	−1.27	1.19	1.27	0.22	0.46
	Chip44	0.23	0.85	0.78	0.16	0.7
	Chip45	−1.16	1.02	1.18	0.21	0.5
Item reliability			0.97			
Person reliability			0.61			
Item Separation			6.08			
Person separation			1.01

Table 4 presents the analysis of evidence for convergent validity between the CHIP and the Ways of Coping checklist. Only the CHIP family dimension correlated negatively and statistically significantly with the distancing and escape-avoidance dimensions of Folkman and Lazarus’s questionnaire, signifying that the greater the scores of items measuring family integrity, cooperation, and optimistic framing, the lower the scores connected to the distancing and escape-avoidance dimensions.

**Table 4:** Strength of the Spearman correlations between the dimensions of the CHIP and the *Ways of Coping* checklist

	CHIP family	CHIP support	CHIP medical
Confrontation	−.15	−.02	.04
Distancing	−.34*	−.07	.01
Self-control	−.17	−.03	−.05
Social support	−.03	.06	.19
Accepting responsibility	−.13	−.02	−.04
Escape-avoidance	−.28*	−.06	.02
Problem-solving	.03	.04	.09
Positive reappraisal	−.03	−.08	.07

*p < .05

## Discussion

The present study contributes to the area of psychological assessment by adapting and examining the psychometric properties of an instrument for evaluating parental coping to a Brazilian sample. The results of the rating scale analyses of the Brazilian version of the CHIP scales suggests that all items satisfied the fundamental requirements of the Rasch model, in accordance with the infit and outfit criteria used for item fit to the measurement models. The three subscales of the Brazilian version of the CHIP revealed themselves to be unidimensional, as indicated by the residual principal component analysis. Although local dependence and factor loadings below .4 at principal component analysis were violated on two items of each scale, the high correlations may be explained by the items’ content similarity. For example, item Chip42 (“explaining our family situation to friends and neighbors so that they understand”) and Chip44 (“chatting with other people/parents in the same situation”) have a clear communicative intention directed to people other than family members for support. It is important to notice that this characteristic is theoretically a part of a general support construct but the evidence here is that these two items share some variance in common that is additive to the overall support measure and not a different dimension.

It is understood that this data can be partly seen as a result of limitations of the study sample. The convenience criterion was used to recruit the participants, so the sample was composed of parents of children with different health problems, levels of dependence, and need for care. The study also included parents of children with acute diseases of transitory course: 33.2% of children did not receive special care and 59.1% did not require intensive medical care in the year prior to the study. Through the responses of the parents of children with less severe conditions, this sample bias may have influenced the scores on items such as Chip42 and Chip44. On this issue, it is thought that, although there have been violations of the assumptions of the analysis, these items should be kept in the scale until additional analyses are carried out. Further random and more uniform sample studies may help to decide whether to exclude or include additional items that map onto a wider range of coping patterns.

These results, while preliminary, suggest that the scores of the three subscales seem appropriate to the measurement model, corroborating the theoretical model of the original instrument (McCubbin 1987). In general, little variability in scores was observed, and in the case of some items, no participant gave the lowest score (i.e., 0). This can also be checked in Table 3, where, for the family and medical scales, the person separation index is below 2, indicating that only one distinguishable stratum can be found in the sample. This indicates that items that are more difficult are needed in order to differentiate participants more precisely. The person reliability indexes (both bellow .65) also corroborate these findings.

The support scale had a person separation of 2.22 suggesting that items are discriminating the sample more effectively in comparison with the medical and family scale. Also, the person reliability of the support scale was .83 indicating that items’ range of difficulty was able to discriminate the sample, yielding reliable distinctions among participants.

Many reasons may be interrelated to the pattern found in the family and medical scales. This could be due to (1) the fact that the greater part of the data collection was done in person and in specialized centers for treatment of individuals with health problems—in this sense, the scores may have been influenced by social desirability (it is expected that the parents of children with problems make greater use of coping strategies) and by the fact that they will be mobilized regarding the care of the children (thereby using a great number of coping strategies)—and (2) the homogeneity in the distribution of the item difficulty—given that there was no evidence of items that require a large amount of the latent trait for endorsement of the highest categories like “extremely helpful,” many of the responses were fell into the uppermost categories. As result, the decision to combine the two response categories seemed useful since no discrimination was obtained with those categories. The same procedure was carried out in a study developed in India where the authors rescored the response categories by collapsing categories 2 and 1 into a single new category upon which subsequent psychometric property analysis of the Indian CHIP was performed (Gothwal et al. 2015.

Nevertheless, the item separation and reliability indexes were acceptable for all of the scales, indicating that the scales themselves have a reproducible item difficulty ordering, implying that the person sample was large enough to confirm the item difficulty hierarchy.

Correlation results of person measure parameters of the three CHIP scales with the Ways of Coping scores showed that only the family scale had a weak negative correlation with the distancing and the escape-avoidance dimensions of Ways of Coping. This indicates that the greater the tendency for family integration, cooperation, and optimistic definitions, the lower the tendency of the participants to avoid resolving problems by means of distancing, avoidance, or escape.

Concerning these points, some studies show that in clinical groups, coping strategies that focus on emotion (such as in the case with distancing and escape-avoidance) are less adaptive and helpful and, therefore, may be less efficacious for reducing tension and stress (e.g., Dabrowska and Pisula 2010), although this is controversial. However, it is noteworthy that the greater part of the dimensions of the measurements used for the convergent validity test were not correlated. This finding can be explained by the facts that (1) these instruments have different dimensions with regard to content and quantity; (2) one instrument is specific to the area of health while the other is not; and (3) the measurements measure different modalities of the same theoretical construct, i.e., while Ways of Coping investigates to what degree coping strategies are used, the CHIP evaluates the judgment of people as to how helpful they are in resolving conflicts. This means that the participants of this study could have employed a certain strategy, but not have considered it helpful. In these conditions, the lack of correlation between the majority of the dimensions of the measurements employed in the investigation of the convergent validity is understandable. Another explanation is that the two subscales of both instruments measure slightly different constructs of coping, thus do not provide much additional validation of the CHIP. As such, this psychometric property stands out as needing further investigation in future studies.

Further important limitations of the present research deserve to be highlighted. In general, the sample size was reduced vis-a-vis the type of analysis done. Furthermore, a convenience sample was used (as opposed to a probabilistically selected one). This implies selection bias. For example, the unbalanced set of respondents (i.e., mothers vs. fathers): the majority were mothers.. In addition, there was no exploration of how differential items functioned between different demographic groupings (e.g., gender, age), which could be addressed in future studies.

## Conclusions

Although the items seem to capture the experiences of a small subset of the population, the implications of the present study are important for professionals and researchers working with parents of individuals with health problems in Brazil. In the area of psychological assessment, it presents preliminary psychometric evidence that CHIP can be used for measuring different dimensions of coping and that it may be helpful for planning services for families of people with health problems. Also, the results enabled verification of the relative difficulty of each item throughout the latent continuum and comparison with the distribution of person parameters (item/person maps, Figs. 1, 2, and 3), which would not have been possible using traditional psychometric statistical analyses. Future studies may extend the scale by including additional items that map onto a wider range of parental experiences when coping with the child’s health problems.

## Additional file

Additional file 1: Inventário de *Coping* parental – Área da Saúde. (DOC 87 kb)

## Abbreviations

ASD: Autism spectrum disorder
CHIP: Coping Health Inventory for Parents
