# Supplementary material for: Brazilian study of adaptation and psychometric properties of the Coping Health Inventory for Parents
Source: Psicol Reflex Crit. 2017 May 11;30:10. doi: 10.1186/s41155-017-0065-9 (PMC6964195; doi:10.1186/s41155-017-0065-9)
Supplement: Supplementary file 1 — Inventário de Coping parental – Área da Saúde. (DOC 87 kb) [file 41155_2017_65_MOESM1_ESM.doc]

Additional file 1

**Inventário de *Coping* parental – Área da Saúde**

**Para cada estratégia que você utilizou para lidar com problemas relativos ao seu filho, lembre o quão útil ela foi. Marque um X na opção que melhor representa sua resposta para cada uma das questões. Utilize as seguintes categorias de resposta: Extremamente útil; Moderadamente útil; Minimamente útil; e Não útil.**

Para cada estratégia que você não utilizou, lembre o motivo, escolhendo uma das opções: “Escolhi não utilizar” ou “Não foi possível”. Nenhuma questão deverá ficar sem resposta.

|  | **Extremamente útil** | **Moderadamente útil** | **Minimamente útil** | **Não útil** | **Escolhi não utilizar** | **Não foi possível** |
| --- | --- | --- | --- | --- | --- | --- |
| 1. Acreditar que meu(s) filho(s) vai (vão) melhorar |  |  |  |  |  |  |
| 2. Investir em meu(s) filho(s). |  |  |  |  |  |  |
| 3. Realizar diversas atividades com meu(s) filho(s). |  |  |  |  |  |  |
| 4. Acreditar que as coisas vão sempre dar certo. |  |  |  |  |  |  |
| 5. Dizer a mim mesmo(a) que eu tenho muitas coisas pelas quais agradecer. |  |  |  |  |  |  |
| 6. Construir uma relação mais próxima com o meu(minha) esposo(a). |  |  |  |  |  |  |
| 7. Conversar sobre sentimentos pessoais e preocupações com meu esposo(a). |  |  |  |  |  |  |
| 8. Realizar diversas atividades com meus familiares. |  |  |  |  |  |  |
| 9. Acreditar em Deus. |  |  |  |  |  |  |
| 10. Cuidar bem, em casa, dos instrumentos de cuidado básico à saúde. |  |  |  |  |  |  |
| 11. Acreditar que o meu filho está recebendo o melhor cuidado médico possível. |  |  |  |  |  |  |
| 12. Tentar manter a estabilidade familiar. |  |  |  |  |  |  |
| 13. Fazer coisas juntos, como uma família (envolvendo todos os membros da família). |  |  |  |  |  |  |
| 14. Confiar em meu(minha) esposo(a) (ou ex-esposo(a)) para ajudar a apoiar a mim e a meu(s) filho(s). |  |  |  |  |  |  |
| 15. Mostrar que eu sou forte. |  |  |  |  |  |  |
| 16. Obter ajuda de outros membros da família com as tarefas domésticas. |  |  |  |  |  |  |
| 17. Ter o meu(minha) filho(a) que tem problema de saúde atendido na clínica/hospital regularmente. |  |  |  |  |  |  |
| 18. Acreditar que o centro médico/hospital está interessado no melhor para a minha família. |  |  |  |  |  |  |
| 19. Incentivar o(s) filho(s) que tem (têm) problemas de saúde a ser mais independente(s). |  |  |  |  |  |  |
| 20. Participar em atividades sociais (festas, etc.) com os amigos. |  |  |  |  |  |  |
| 21. Conseguir se afastar das tarefas e responsabilidades domésticas para ter algum descanso |  |  |  |  |  |  |
| 22. Ficar longe sozinho. |  |  |  |  |  |  |
| 23. Comer. |  |  |  |  |  |  |
| 24. Dormir. |  |  |  |  |  |  |
| 25. Permitir-me ficar com raiva. |  |  |  |  |  |  |
| 26. Comprar presentes para mim e/ou para outros membros da família |  |  |  |  |  |  |
| 27. Concentrar-se em passatempos (arte, música, corrida, etc.) |  |  |  |  |  |  |
| 28. Trabalhar, ter um emprego fora de casa. |  |  |  |  |  |  |
| 29. Tornar-me mais autoconfiante e independente. |  |  |  |  |  |  |
| 30. Manter-me em forma e bem arrumado(a) |  |  |  |  |  |  |
| 31. Conversar com alguém (que não seja psicólogo ou médico) sobre como eu me sinto. |  |  |  |  |  |  |
| 32. Engajar em relacionamentos e amizades que me ajudam a sentir-me importante e apreciado. |  |  |  |  |  |  |
| 33. Divertir os amigos em nossa casa. |  |  |  |  |  |  |
| 34. Investir tempo e energia em meu trabalho. |  |  |  |  |  |  |
| 35. Sair regularmente com meu esposo(a). |  |  |  |  |  |  |
| 36. Construir relacionamentos próximos com as pessoas. |  |  |  |  |  |  |
| 37. Desenvolver-me como pessoa. |  |  |  |  |  |  |
| 38. Conversar com outros pais que passam pelo mesmo tipo de situação e aprender sobre as experiências deles. |  |  |  |  |  |  |
| 39. Conversar com a equipe médica (enfermeiros, assistente social, etc.), quando visitamos o centro médico. |  |  |  |  |  |  |
| 40. Ler sobre como as outras pessoas na mesma situação lidam com as coisas. |  |  |  |  |  |  |
| 41. Ler mais sobre o problema de saúde que me preocupa. |  |  |  |  |  |  |
| 42. Explicar nossa situação familiar para amigos e vizinhos para que eles entendam. |  |  |  |  |  |  |
| 43. Saber que os tratamentos médicos prescritos para a(s) criança(s) são seguidos em casa diariamente. |  |  |  |  |  |  |
| 44. Conversar com outras pessoas/pais na mesma situação. |  |  |  |  |  |  |
| 45. Conversar com o médico sobre as minhas preocupações sobre meu(s) filho(s) com problema de saúde. |  |  |  |  |  |  |
